# Medical short text classification via Soft Prompt-tuning

**DOI:** 10.3389/fmed.2025.1519280

**Published:** 2025-04-14

**Authors:** Xiao Xiao, Han Wang, Feng Jiang, Tingyue Qi, Wei Wang

**Affiliations:** ^1^Department of Ultrasound, The Affiliated Hospital of Yangzhou University, Yangzhou University, Yangzhou, China; ^2^Department of Information Engineering, Yangzhou University, Yangzhou, China; ^3^Department of Ultrasound, The First Affiliated Hospital of Wannan Medical College, Wuhu, China; ^4^Department of Radiology, The Affiliated Hospital of Yangzhou University, Yangzhou University, Yangzhou, China

**Keywords:** medical short text, short text classification, prompt-tuning, soft prompt, NLP

## Abstract

In recent decades, medical short texts, such as medical conversations and online medical inquiries, have garnered significant attention and research. The advances in the medical short text have profound implications in practical applications, particularly for classifying in-patient discharge summaries and medical text reports, leading to improved understandability for medical professionals. However, the challenges posed by the short length, professional medical vocabulary, complex medical measures, and feature sparsity are further magnified in medical short text classification compared to general domains. This paper introduces a novel soft prompt-tuning method designed specifically for medical short text classification. Inspired by the recent success of prompt- tuning, which has been extensively explored to enhance semantic modeling in various natural language processing tasks with the appearance of GPT-3, our method incorporates an automatic template generation method to address the issues related to short length and feature sparsity. Additionally, we propose two different strategies to expand the label word space, effectively handling the challenges associated with specialized medical vocabulary and complex medical measures in medical short texts. The experimental results demonstrate the effectiveness of our method and its potential as a significant advancement in medical short text classification. By addressing issues related to short text length, feature sparsity, and specialized medical terminology, it offers a promising advancement toward more accurate and interpretable medical text classification.

## 1 Introduction

In the past few years, short texts have been posted at unprecedented rates, which emphasizes the significance of learning tasks and also highlights the challenges stemming from the vast feature space (1). Different from traditional documents, short texts present considerable obstacles to the effectiveness of mainstream text classification solutions due to their short length, feature sparsity, and high ambiguity. The advances in short text classification have significant implications in practical applications including medical-aided diagnosis (2), thereby necessitating an urgent need to comprehend and address the characteristics of short texts.

With the rapid development of the Internet, online medical inquiries have garnered significant attention in the real world. These inquiries are presented in narrative formats that retain the characteristics of ambiguity and informality (3). Within these medical short texts, the presence of professional medical vocabulary and complex medical measures varies depending on the responses provided to different users. Moreover, the use of abbreviations and diverse forms of expression complicates the discovery of underlying patterns using conventional short text methods (4). Additionally, due to the requirements on professional expert knowledge, it is time-consuming and labor-intensive to collect enough labeled data for medical short text, which remains prohibitively expensive and impractical to learn in existing deep learning methods (5).

In recent decades, the research paradigm of medical short text classification has transformed, shifting from early feature engineering-based methods to the adoption of neural networks, which have been extensively applied and demonstrated superiority in this task. Prominent deep neural networks, such as Convolutional Neural Network (CNN) (6), Recurrent Neural Network (RNN) (7), Long Short-Term Memory (LSTM) (8), and autoencoder (9), have exhibited impressive performance in learning more abstract and higher-level representations for medical short texts. For instance, (10) proposed the optimized TextCNN model for Chinese medicine text classification, which yielded highly promising results. Recently, Pre-trained Language Models (PLMs) such as BERT (11), RoBERTa (12), T5 (13), and GPT (14) have emerged as powerful tools for language understanding and generation. To leverage the rich knowledge embedded in PLMs for various natural language processing (NLP) tasks, the fine-tuning method, coupled with an additional classifier, has been widely employed and achieved remarkable performance across various downstream tasks, including medical short text classification (15).

More recently, inspired by the success of GPT-3, prompt-tuning has gained extensive attention to enhance semantic modeling in various Natural Language Processing (NLP) tasks (16, 17). In prompt-tuning, hand-crafted or auto-generated templates are employed to formalize downstream NLP tasks into cloze-style filling tasks. For instance, in the context of medical short text classification, given the input sentence *x* as “Which department of the hospital should be registered for epilepsy?,” prompt-tuning with a hand-crafted template wraps it into “A problem for [MASK]: *x*.” The probability of different topic words, such as “Neurology” or “Endocrinology,” is then calculated to fill the “[MASK]” token. Remarkably, without the need for fine-tuning on large volumes of labeled data for the downstream task, prompt-tuning has exhibited promising performance, even in few-shot or zero-shot learning scenarios. Despite the effectiveness of prompt-tuning methods with hand-crafted templates in various NLP downstream tasks, their construction remains time-consuming and labor-intensive, requiring substantial human effort. Moreover, poorly designed templates can degrade model performance. More recently, soft prompt-tuning methods have been explored (18). Unlike hand-crafted templates, soft prompts are continuous representations, typically encoded as vectors, that can be optimized during training to achieve better performance.

In this paper, we present a novel method for Medical short text classification via Soft Prompt-tuning (short for MSP). Our method aims to address the challenges posed by professional medical vocabulary and complex medical measures in medical short texts. Specifically, we construct the mapping from the expanded label words (e.g., breast, sterility, obstetrics, Cervical diseases, gynecologist, etc.) to their corresponding categories (e.g., gynecology and obstetrics) in prompt-tuning. This mapping, referred to as “verbalizer,” has been proven effective in reducing the discrepancy between the text and label spaces (19). In our method, we propose two strategies, i.e., Concepts Retrieval and Context Information, to construct the verbalizer, each capturing different characteristics of the expanded words. The integration of these two strategies yields the final verbalizer, which significantly enhances the accuracy of classification. Moreover, to accommodate the requirements for large-scale labeled training datasets, our MSP method is grounded in soft prompt-tuning. Soft prompt-tuning incorporates the vector of the input sentence, the mask, and the soft tokens, enabling the model to achieve robust performance even in few-shot scenarios. The experimental results on online medical inquiries demonstrated the effectiveness of our MSP method compared to other state-of-the-art methods for medical short text classification. The contributions of our method can be summarized as follows:

- Our MSP is a novel medical short text classification method based on prompt-tuning. Compared with existing methods, MSP can achieve better performance even in few-shot scenarios.
- The verbalizer is constructed for the professional medical field, and two strategies are employed to capture different characteristics of the expanded words. The integration of these strategies is utilized as the final verbalizer.
- The experimental results on real-world online medical inquiries demonstrated that our MSP obtains new state-of-the-art results compared to other deep neural networks and fine-tuned PLMs methods.

## 2 Related work

In this section, we review the related work on medical short text classification and prompt-tuning in detail, respectively.

### 2.1 Medical short text classification

The medical short text has garnered significant attention and research in recent decades, and the advancements in this area have had far-reaching implications in various practical applications, such as medical-aided diagnosis (20) and online medical inquiries (21). Specifically, medical short text classification focuses on predicting accurate labels for texts with limited length. For instance, in the context of online medical inquiries, both the problem and its corresponding answer usually consist of fewer than 20 words (22).

In recent years, deep neural networks have demonstrated remarkable performance in medical short text classification tasks, attributed to their ability to learn abstract and higher-level feature representations. For instance, Kim proposed the TextCNN model, which achieved substantial performance in sentence-level classification tasks by training a CNN with a single layer on top of pre-trained word vectors (23). The model kept the word vectors static while tuning and learning the parameters of only one CNN layer. In the domain of medical short text classification, Li et al. introduced the convolutional layer to extract features from sentences and utilized bidirectional gated recurrent unit (BIGRU) to learn both preceding and succeeding sentence features. Additionally, an attention mechanism was employed to obtain sentence representations with important word weights (5). BIGRU and the attention mechanism were also leveraged for document representation learning, serving as both encoding and decoding layers. (24) proposed the incorporation of word-cluster embedding into deep neural networks to address the problem of semantic feature scarcity and ambiguity. The method involved hierarchical agglomerative clustering to cluster word embeddings in the semantic space. The resulting cluster center vectors served as potential theme information, and CNN and LSTM were employed for classification using the cluster-based features. In the context of online medical inquiries, (3) proposed a three-stage hybrid system for classification. The system combined a regular expression-based classifier with attentive bidirectional Long Short-Term Memory (ABLSTM) to achieve high classification results. ABLSTM was introduced to extract feature words of high quality, and the word weights were then utilized in constructing regular expression-based text classifiers. Furthermore, (2) proposed a method for Chinese electronic medical record classification based on an improved capsule network. In this approach, Chinese medical short texts were initially processed using an LSTM network, followed by the utilization of the Capsule network to achieve improved performance.

Recently, fine-tuned Pre-trained Language Models (PLMs), including BERT (11), ALBERT (25), RoBERTa (12), T5 (13), and GPT (14), have emerged as powerful tools for leveraging rich knowledge in NLP downstream tasks. Through fine-tuning PLMs with specific downstream tasks, latent information can be learned, leading to tremendous success in various NLP tasks, including medical short text classification. Given the exceptional performance of fine-tuned PLM methods, it is widely acknowledged that training new models from scratch can be avoided. For instance, (26) proposed a Knowledge Graph enhanced multiType text BERT method for medical text classification. This approach integrates the medical knowledge graph to extract standard entity names from medical text. Initially, the same BERT-Encoder is employed to process multi-type text, and then multiple encodings are concatenated to form the representation matrix. Different types of pooling layers are explored for information summation. (27) proposed an optimal deep learning model based on BERT and hyperparameter selection for medical text classification. The BERT model is used to learn the feature representations of medical texts, followed by the utilization of the Particle Swarm Optimization (PSO) algorithm for selecting hyperparameters for the deep learning classifier. (4) proposed an ALBERT-based fusion Kalman-filter model to address word-level and sentence-level noises for medical short texts. They first employ a sliding window scheme to handle the coupling relationships of large sequences and then use the fusion block to integrate features of multiple segment sequences. The ALBERT architecture with four iterative encoder layers is leveraged as PLMs for word embedding learning. (28) proposed a Traditional Chinese Medicine (TCM) text classification method based on RoBERTa. They fine-tuned the RoBERTa model with TCM medical records data and then tokenized the classified sample data using the Tokenizer based on the pre-trained RoBERTa model. Wang et al. propose the use of a discriminative pre-training language model called ERNIE-Health for classifying medical texts. Specifically, the authors attempt prompt tuning based on a multi-token selection task, wrapping the original text in a template into a new sequence where category labels are replaced with [UNK] tokens. The model is then trained to compute the probability distribution of candidate categories. Adapting prompt-tuning methods designed primarily for English to Chinese text classification tasks presents challenges (29). Li et al. introduce Knowledge Enhanced Multi-Token Prompt Tuning (KMPT). The implementation involves initially using multiple tokens as label words with complete Chinese semantics. Subsequently, external knowledge is utilized to expand the set of label words, improving coverage and reducing bias (30).

### 2.2 Prompt-tuning

Despite the success of fine-tuning PLMs, recent studies have identified a critical challenge: the significant gap in objective forms between pre-training and fine-tuning, which limits the exploitation of knowledge in PLMs. To tackle this issue, prompt-tuning has emerged with inspiration from GPT-3 for improving semantic modeling across a wide range of NLP tasks (31). Prompt-tuning involves inserting input statements into natural language templates and adjusting the mask model to transform tasks into cloze-style filling tasks (17). The prompt-tuning has been widely explored and applied with tremendous success in various downstream NLP tasks, including information extraction (32), question answering (33), text generation (34), and text classification (35).

The primary components of prompt-tuning include a template and a set of label words. The template serves as a background description of the current task, while the label words consist of the high-probability vocabulary predicted by PLMs in the given context. Initially, hand-crafted templates, which involve discrete prompts manually specified and kept unchanged during training, were proposed and applied. For instance, (36) encoded prior knowledge of a classification task into rules and decomposed it into sub-tasks, combining human-picked sub-prompts for the final classification tasks. (37) introduced learning discrete prompts through continuous optimization, which achieved notable performance in both image generation and language classification tasks. In the context of relation extraction, (32) incorporated knowledge among relation labels into prompt-tuning. The method involved injecting learnable virtual answer words into semantic knowledge to represent relation labels and then optimizing the representations with structured constraints rather than relying on entity-type annotations.

Although hand-crafted templates have shown substantial success in various NLP tasks, their construction is often time-consuming and labor-intensive, and inappropriate templates may lead to substandard model performance. To address this, more recently, automatic-generated template methods, i.e., soft templates, have been explored (18, 38). In contrast to hard templates, soft templates are continuous prompts, usually presented as vectors, that can be continually optimized during training to yield optimal results. For example, (39) proposed to learn a mixture of soft prompts to extract relational knowledge from language models, which has shown that continuous vectors can achieve impressive performance compared to “hard prompts.” (40) introduced an automatic prompt generation method that achieved promising results in Natural Language Understanding tasks by identifying the most suitable template for downstream tasks and incorporating learnable vectors into the template while continually optimizing it during training. Additionally, (41) proposed a transfer learning method based on soft prompts, where a prompt is first trained on one or more source tasks, and then the resulting prompt is utilized to initialize the prompt for the downstream tasks.

In addition to the template used in prompt-tuning, the mapping from label words to categories, known as the verbalizer, has been demonstrated to effectively address the discrepancy between text and label space (19). There have been efforts to create hand-collected verbalizers for various NLP downstream tasks. For instance, (42) proposed to use pairs of cloze question patterns and manually designed verbalizers to leverage the knowledge contained within PLMs for downstream tasks. However, manually designed verbalizers are heavily influenced by prior knowledge, leading to potential omissions and biases in knowledge expansion. Automatic verbalizer construction methods have been developed to mitigate these issues (43–45). For example, (35) introduced a knowledgeable expansion prompt-learning method for short text classification. This approach incorporates both the short text itself and external knowledge from open Knowledge Bases, such as Probase, to extend the label words space. Several different strategies are employed for automatic verbalizer construction. (46) proposed a method to elicit knowledge from PLMs for constructing verbalizers. In this method, the label information is encoded as prototypical embeddings in the latent feature space, and representations of masked words and prototypical embeddings are calculated for the classification tasks. Furthermore, (47) devised a method to incorporate imprecise knowledge from large unlabelled corpora into verbalizer construction for biomedical text relation extraction. In this method, word and relation word embeddings are learned to infuse entity and relation information, and biomedical domain knowledge constraints are introduced to enhance representations.

## 3 Methodology

**Figure 1:**
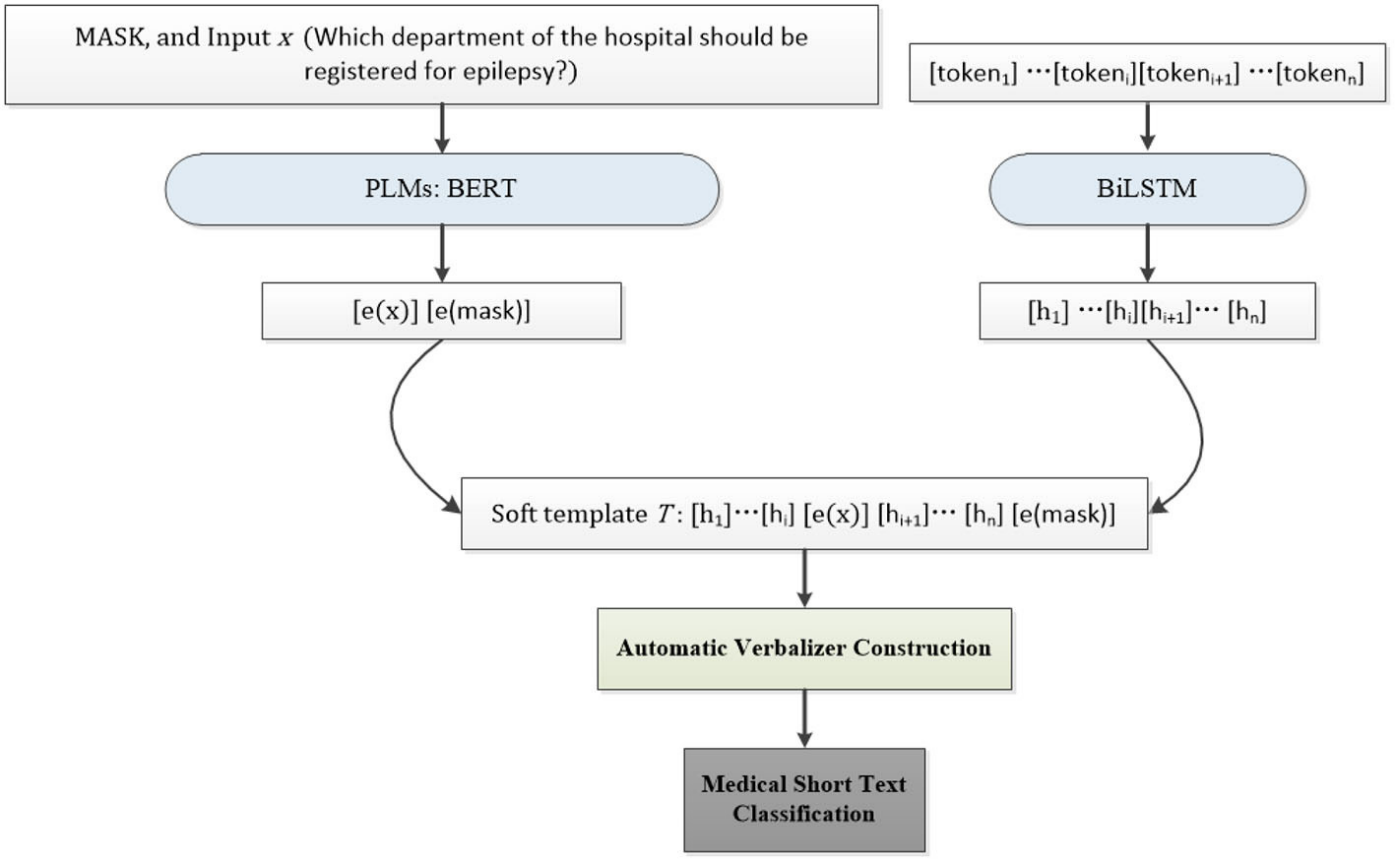
The illustration of our MSP. There are three main components in our method: soft template generation, automatic verbalizer construction, and medical short text classification. Firstly, the mask and the input *x* are mapped into the embedding by PLMs such as BERT in the experiments. The neural network, i.e., BiLSTM in our experiments, is then employed to train soft tokens in the soft template. Secondly, the top *N*_*a*_ concepts concerning the entities in the medical short text itself are retrieved by two strategies for automatic verbalizer construction. Finally, the constructed soft prompt and verbalizer are used to predict the probability of each label word belonging to a special class for medical short text classification.

The whole framework of our MSP is illustrated in Figure 1. In this section, the automatic template generation, verbalizer construction, and medical short text classification are described successively in detail.

### 3.1 Soft template generation

As depicted in Figure 1, the soft template *T* comprises two main components: the soft prompt tokens and the embeddings of the input sentence *x* and MASK. For example, in the aforementioned example, where the task is to predict the topic of the sentence *x*: “Which department of the hospital should be registered for epilepsy?,” the prediction is based on the probability that the topic word, such as “Neurology” or “Endocrinology,” fills the [*MASK*] token.

In the experiments, we utilized BERT as PLMs to learn the embeddings of the input sentence *x* and MASK. The input sentence *x* is represented as *x* = {*x*_0_, …, *x*_*i*_, …, *x*_*h*_}, which is mapped into the embeddings as *e*(*x*) = {*e*(*x*_0_), …, *e*(*x*_*i*_), …, *e*(*x*_*h*_)} by PLMs *e*. Similarly, the mask is mapped into the embeddings as *e*(*mask*). Then the soft tokens in the template are denoted as [token_1_]…[token_*i*_][token_*i*+1_]…[token_*n*_]. In contrast to manually designed templates, the soft template generation involves the use of a BiLSTM as the neural network to train the initial soft tokens, resulting in *h*_1_, …, *h*_*i*_, *hi*+1, …, *h*_*n*_. The soft prompt *T* can then be represented as Equation 1:


(1) $$\begin{array}{l} T=\left\{h_{0},...,h_{i},e\left(x\right),h_{i+1},...,h_{n},e\left(mask\right)\right\} \end{array}$$

where *h*_*i*_ can be formulated as Equation 2:


(2) $$h_{i}=\left({\vec{h}}_{i},{\overset{\leftarrow }{h}}_{i}\right)=\left(\vec{LSTM}\left(h_{0},\vec{h_{i-1}}\right),\overset{\leftarrow }{LSTM}\left(h_{i+1},\overset{\leftarrow }{h_{n}}\right)\right)$$

The soft prompt *T* enables us to discover better continuous prompts for enhancing the performance of downstream tasks. Finally, the loss function *L* for the short text function with respect to *h*_*i*_ can be formulated as Equation 3:


(3) $$\hat{h}=\underset{h_{i}}{\text{arg min }}L\left(M\left(x,mask\right)\right)$$

### 3.2 Automatic verbalizer construction

In prompt-tuning, verbalizer refers to the mapping from the expanded label words (e.g., breast, sterility, obstetrics, Cervical diseases, gynecologist, etc.) to their corresponding categories (e.g., gynecology and obstetrics), which has been empirically proven to effectively reduce the discrepancy between text and label spaces, thereby enhancing the performance of downstream tasks (19). In contrast to previous methods that directly search concepts in large knowledge bases using category names (44, 45), we propose two different strategies, namely Concepts Retrieval and Context Information, to expand the label words from the short text itself. Each strategy captures different aspects of the characteristics of the expanded words, and these words are subsequently integrated into the final verbalizer. Below are the details of the two strategies:

#### 3.2.1 Concepts retrieval

In previous verbalizer construction methods, such as those mentioned in (44) and (45), the expanded label words were identified in large knowledge bases through semantic similarity calculation. However, these methods not only yielded unsatisfactory performance in medical short text classification but also proved to be time-consuming and labor-intensive. To tackle this issue, in this paper, we first retrieved concepts related to entities mentioned in the medical short text from an open knowledge base, such as Probase,^1^ which provides the probability of each entity belonging to a particular concept. This novel approach, termed Concepts Retrieval, allows us to address the challenges posed by professional medical vocabulary and complex medical measures in medical short text classification. Moreover, it enables us to avoid searching the entire knowledge base and focus solely on retrieving professional medical concepts by leveraging Probase for probabilities ranking. To be more specific, we retrieved *N*(*v*) concepts from Probase ranked by their probabilities. Subsequently, we introduced category names *y* (such as “Neurology” and “Endocrinology” in medical text datasets as anchor words. The distance *dist*(*V*_*y*_, *y*) between each expanded label word and the category name *y* was calculated in the embedded space. In our experiments, we selected the top *N*_*a*_ words, excluding morphological derivations of *y*. In the experiments, *N*_*a*_ = 15 is used for Concepts Retrieval strategy in the experiments.

#### 3.2.2 Context information

To expand words while incorporating context information from the words preceding and following the masked word, we employed PLMs such as BERT in our experiments, as opposed to traditional N-gram language modeling. However, due to that BERT is a non-autoregressive language model, it cannot directly compute the likelihood of a sentence. To address this, we introduced a symmetric window of size *c* around the “[MASK]” word as the context. Let *W* =…*w*_−*c*_, …*w*_−1_, *w, w*_1_, …*w*_*c*_, … represent the context of the masked word *w*. We then masked each *w*_*i*_ in *W* from front to back and fed it into the BERT model to compute the loss of *w*, as expressed in Equation 4:


(4) $$\begin{array}{l} L\left(w_{i}\right)=-\underset{v_{i}\in V}{\sum }1\left\{v_{i}=w_{i}\right\}\times \log p\left(v_{i}=w_{i}|W_{\backslash w_{i}}\right) \end{array}$$

where *V* represents the set of words in the vocabulary, 1· is the indicator function, and *p*(*v*_*i*_ = *w*_*i*_|*W*_\_*w*__*i*__) is the BERT prediction distribution that is conditioned on *W* excluding *w*_*i*_. The total loss of *W* is then computed as the average of loss for each word *w*_*i*_, which can be represented as Equation 5:


(5) $$\begin{array}{l} L\left(W\right)=\frac{1}{2c+1}\overset{i=c}{\underset{i=-c}{\sum }}L\left(w_{i}\right) \end{array}$$

Finally, all the expanded words are sorted based on their corresponding sequence loss *L*(*W*). In our experiments, we set *c* to 5 and discarded words with higher loss. Similar to the “Concepts Retrieval” strategy, we finally selected *N*_*a*_ words among all the predicted words to construct the verbalizer, and *N*_*a*_ = 15 is also used for context information strategy in the experiments.

### 3.3 Medical short text classification

Once we have constructed the final verbalizer for medical short text, the predicted probability for each label word needs to be mapped to a specific category. This mapping process can be represented by an objective function denoted as *g*. Since we assume that each word in the final verbalizer contributes equally to the prediction, we use the average of the predicted scores for text classification. Specifically, *g* can be calculated as Equation 6:


(6) $$\begin{array}{l} \arg\underset{y\in Y}{\max}\frac{1}{\left|V_{y}\right|}\underset{v\in V_{y}}{\sum }p\left(\left[\text{MASK}\right]=v|x_{p}\right) \end{array}$$

where *V*_*y*_ represents the set of label words corresponding to the label *y*, and |*V*_*y*_| denotes the cardinality of *V*_*y*_. The function *p*([MASK] = *v*|*x*_*p*_) computes the probability of the label word *v* given the input text *x*_*p*_.

## 4 Experiments

### 4.1 Datasets

To validate the effectiveness of our proposed MSP, we utilized web crawling techniques to acquire a certain amount of data from the Chinese medical conversation dataset. This enabled us to derive the symptom diagnosis classification dataset and the gynecological multi-classification dataset, which were then subjected to experimental analysis. Below is a comprehensive depiction of the two datasets:

- Symptom dataset. This dataset contains contents of eight categories, namely, infectious diseases, proctology, orthopedics, respiratory, andrology, burn, cardiovascular, and plastic surgery, crawled from the Internet, with a total of 80,000 training sets and 4,000 test sets of original data.
- Gynecology dataset. This dataset contains contents of the infertility department, obstetrics department, and gynecology department under the gynecology category crawled from the network, with a total of 45,000 original data training sets and 2,100 test sets.

### 4.2 Compared methods

The following methods including deep neural networks and fine-tuned PLMs models are utilized as compared methods.

- Regular Prompt-tuning (PT) (16): It employs hand-crafted templates and label words to form the prompt, along with an ensemble model to annotate an unlabeled dataset, To ensure fairness we use the same sample template for the same dataset.
- TextCNN (23): The CNN architecture is utilized for the task of text classification. In particular, the text undergoes preliminary word segmentation, followed by passing through a convolutional and pooling layer in succession, and the outcome is then passed through an external softmax classifier to classify the text.
- ERNIE (48): By improving the classical PLMs like BERT, the knowledge and linguistic semantic information are integrated to enhance the representation of text semantics, which is more suitable for Chinese natural language processing tasks.
- P-tuning (40): It proposes to learn continuous prompts instead of hand-crafted prompt by inserting trainable variables into the embedded input.

### 4.3 Experiment setting

#### 4.3.1 Training data

To simulate the situation of data scarcity, we conducted experiments using 10-shot, 15-shot, and 20-shot sampling methods to evaluate the effectiveness of our proposed MSP. Here, we provide a detailed account of the training samples used in the experiments. For each K-shot methodology discussed in this paper, along with PT and P-tuning, K sample data from each class were extracted from the original training set to form small-sized training sets. Additionally, another K sample data from each class were extracted to create the corresponding test sets. As the selection of training and verification sets with small samples introduced variability, we executed three random sampling experiments, and the final experimental results were averaged over these instances of random sampling.

Regarding the ERNIE and TextCNN models' performance, we conducted manual sampling by handpicking different numbers of training samples. This allowed us to compare the quantities of 10-shot, 15-shot, and 20-shot samples utilized in the proposed method. Specific sample figures are presented in Table 1.

**Table 1:** The detail of training data of different models.

**Dataset**	**ERNIE**	**TextCNN**	**PT/P-tuning/Ours**
Symptom	2,240/2,400/2,800	4,000/4,400/4,800	80/120/160
Gynecology	1,350/1,380/1,440	720/750/840	30/45/60

#### 4.3.2 Parameter settings

The detailed experimental configurations are as follows: We use Python 3.6 based programming environment on Linux as our foundation. For both datasets, the batch size was set to 64. Considering the dataset distribution, the Symptom diagnosis dataset was trained for a total of 10 epochs, while the Gynecological multi-classification dataset was trained for 20 epochs. The model's learning rate was set to 0.0003 with an AdamW optimizer. The hidden size was set to 200, and a dropout rate of 0.5 was applied and weight decay was set to 0.01, while the pre-training language model parameters were frozen during the training process. To ensure fairness, we used the same experimental settings for all compared methods based on prompt-tuning, such as Regular Prompt-tuning: PT, p-tuning. BERT was used as the backbone PLM, and its implementation was based on the Hugging Face Transformer Library for the main methods.

### 4.4 Experimental results

**Figure 2:**
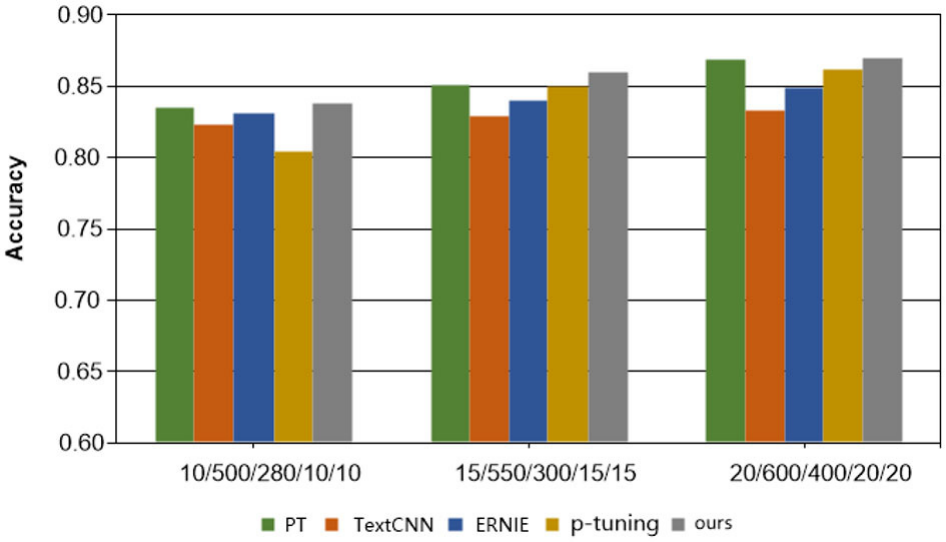
Results of each method on the Symptom dataset.

**Figure 3:**
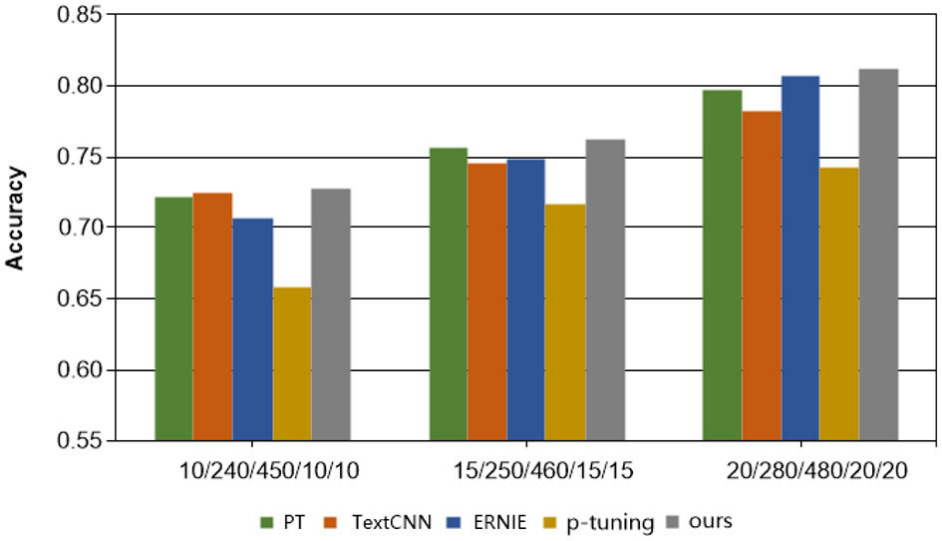
Results of each method on the Gynecology dataset.

Table 2, accompanied by Figures 2, 3, provides a comprehensive analysis of the experimental findings. Based on the experimental results, the following conclusions can be drawn. Firstly, the accuracy of all methods has improved as the number of training samples increases. This indicates that increasing the number of samples in few-shot learning is beneficial for enhancing model performance. Notably, the proposed method exhibits better experimental results compared to the other four comparison methods on all cases in terms of overall classification efficacy.

**Table 2:** The performance of accuracy (%) on all two datasets.

**Dataset**	**Method**	**Training data and the accuracy (%) results**		
		**10/500/280/10/10**	**15/550/300/15/15**	**20/600/400/20/20**
Symptom	PT	83.35±1.57	85.17±1.51	86.87±1.62
	TextCNN	82.28±1.82	82.82±1.65	83.38±2.94
	ERNIE	83.38±5.10	84.54±3.82	85.18±5.30
	p-tuning	80.39±0.98	84.94±0.96	86.25±0.46
	Ours	**83.78**±0.61	**85.67**±082	**86.93**±0.63
		**10/240/450/10/10**	**15/250/460/15/15**	**20/280/480/20/20**
Gynecology	PT	72.38±1.82	75.66±2.53	79.65±1.70
	TextCNN	72.30±2.19	74.52±3.81	78.15±1.92
	ERNIE	70.20±3.55	74.51±5.48	80.73±2.90
	p-tuning	65.79±1.48	71.71±1.06	74.43±0.76
	Ours	**72.60**±0.74	**76.30**±1.05	**81.18**±0.97

The bolder ones mean better.

Secondly, in the Symptom dataset, when employing the PT and P-tuning methods alongside manual templates, the experimental results are significantly better than those of the ERNIE and TextCNN models. However, none of these methods match the performance of our MSP method presented in this paper, validating the effectiveness of the proposed model. On the other hand, the overall effect on Gynecological datasets is not as satisfactory. This can be attributed to the relatively limited content differentiation within the Gynecological classification datasets. Nonetheless, the proposed MSP method shows improvement in classification accuracy by incorporating external knowledge.

Lastly, in the context of Chinese medical dialogue datasets, it is noteworthy that despite the ERNIE model being a pre-trained language model and TextCNN being considered highly adept at Chinese natural language processing tasks, the sample size of the proposed method in this paper is merely 1/20 of those used in the two methods. Despite this, the classification accuracy of the proposed method still surpasses that of these two widely used text classification approaches, highlighting the efficacy of the proposed method, especially in limited data learning scenarios.

### 4.5 Parameter sensitivity

Some important parameters in the experiments often affect the performance, such as learning rate and batch size. In this section, we performed sensitivity experiments on the Symptom dataset under the condition of 20-shot.

#### 4.5.1 Batch size

**Figure 4:**
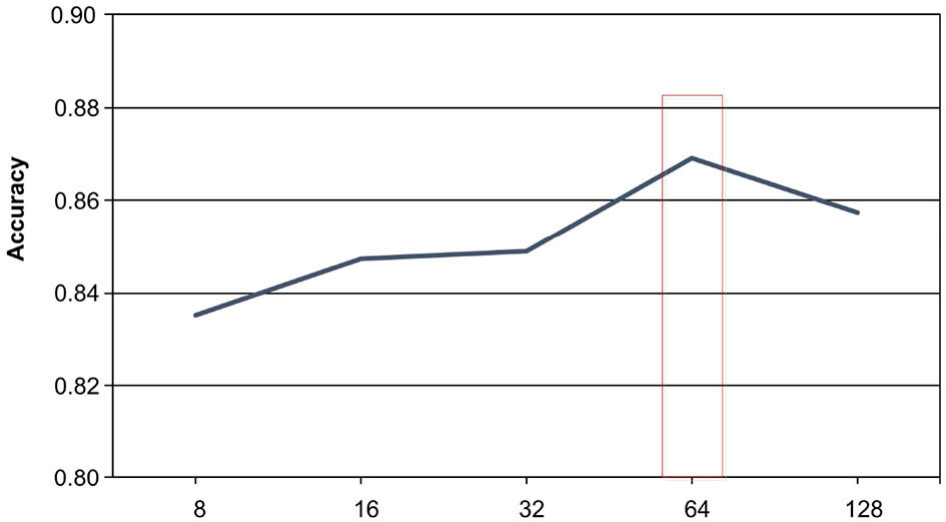
The effect of batch size on experimental results.

The batch size parameter plays a crucial role in determining the performance of the model in the realm of text classification. In our experiment, we conducted a comparison of the model's performance under five different batch sizes: 8, 16, 32, 64, and 128, and analyzed the results. Our empirical findings, as depicted in Figure 4, reveal that the highest accuracy score of 86.91% was attained when the batch size was set to 64, while a suboptimal accuracy score of 83.52% was recorded at a batch size of 8. When the batch size was increased to 128, the accuracy plummeted to 85.72%. We attribute this experimental result to the distinct data distribution patterns learned by the model under different batch sizes. Smaller batch sizes are more susceptible to overfitting due to the relatively limited amount of data processed at each interval. Conversely, excessively large batch sizes tend to complicate the model training process, leading to inefficient learning. Based on our analysis of the Symptom dataset, we conclude that a batch size of 16 represents the most favorable parameter for the model, striking a balance between overfitting and training efficiency.

#### 4.5.2 Learning rate

**Figure 5:**
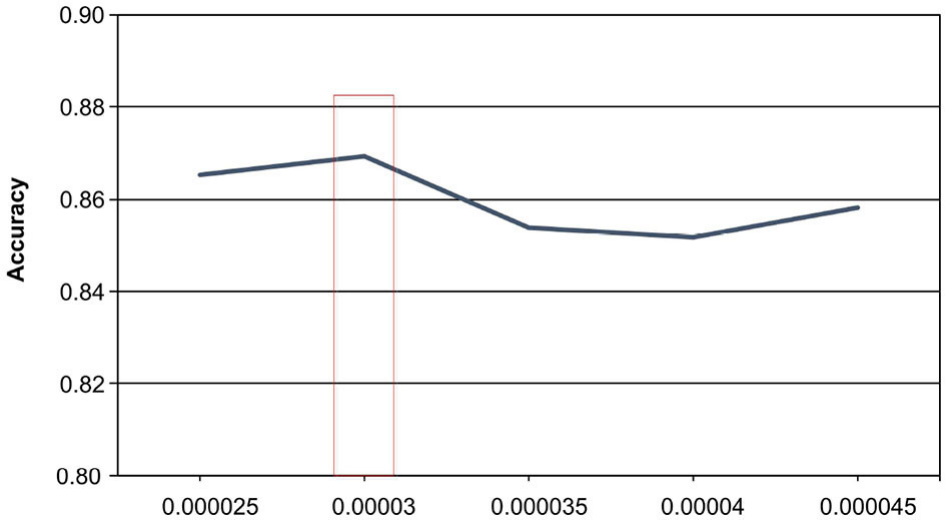
The effect of learning rate on experimental results.

When undertaking the task of medical short text classification, the learning rate parameter plays a crucial role in the optimization process, as it governs the magnitude of weight adjustments during training. The selection of an appropriate learning rate is of utmost importance, given its profound influence on the model's performance. Our analysis of the experimental results, as shown in Figure 5, confirms that the model's performance varies under the influence of the learning rate, with optimal results achieved at a learning rate of 0.00003, reaching an accuracy of 86.91%. Conversely, when the learning rate is set to 0.00004, the model's accuracy is the lowest, measuring at 85.19%.

Achieving optimal performance necessitates the careful selection of an appropriate learning rate. However, it is essential to acknowledge that disparate tasks and datasets may require different learning rates. Hence, conducting experimental testing is imperative to determine the ideal parameter values that yield the best results for a specific task and dataset.

### 4.6 Ablation experiment

**Figure 6:**
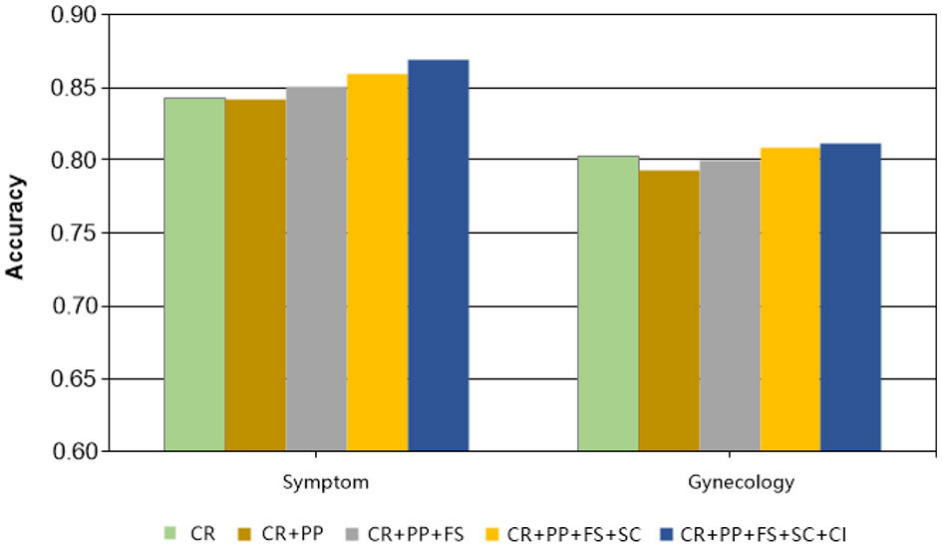
The results of the ablation experiment.

To better exemplify the efficacy of our MSP, we conducted ablation experiments to obtain verbalizers that were introduced at varying degrees. Specifically, instead of engaging two strategies concurrently to construct verbalizer, including Concepts Retrieval (CR) and Context Information (CI). We incrementally incorporated strategies and evaluated experimental performance under 20-shot conditions on two datasets. As Figure 6 evinces, the accuracy of the experiments improved to a certain extent with the integration of diverse strategies. For instance, in the Symptom dataset, the experimental results surged from 84.24% to 86.91 %, representing a 2.67% increase, and there was discernible progress made in the Gynecology dataset as well. The amalgamation of various strategies detailed in this paper yields remarkable outcomes.

## 5 Conclusions

This paper introduces a novel method for medical short text classification with soft prompt-tuning (short for MSP). MSP is proposed to address the problems of professional medical vocabulary and complex medical measures, and it achieved excellent performance even in few-shot scenarios. The soft prompt in MSP comprises the vectors of the MASK and the input sentence learned by BERT, and the soft tokens learned by BiLSTM. Furthermore, two strategies including concept retrieval and context information are adopted for verbalizer construction. Extensive experiments validated the effectiveness of our MSP compared to other neural networks, fine-tuned PLMs, and prompt-tuning methods.

In the future, we will extend the research work of medical short text classification in the following two directions. Firstly, we aim to explore adaptive prompt-tuning techniques that automatically adjust based on data distribution, user intent, or medical domain variations. Secondly, we plan to incorporate more auxiliary information including medical ontologies, clinical guidelines, or electronic health records (EHRs) for improving context understanding and classification accuracy.

## 6 Limitation

In this paper, we have validated the effectiveness of our method on medical short text classification. However, firstly, the constructed verbalizer has not been validated by domain experts or automated knowledge bases, future work should investigate strategies to address this problem. Secondly, the optimization of soft prompts requires additional computational resources compared to traditional hand-crafted prompt-tuning methods. While our method reduces manual effort, further optimizations could be explored to improve computational efficiency.

## Data Availability

The original contributions presented in the study are included in the article/supplementary material, further inquiries can be directed to the corresponding author.

## Footnotes

^1^ https://concept.research.microsoft.com/
